# Knockdown of lectin-like oxidized low-density lipoprotein-1 ameliorates alcoholic cardiomyopathy via inactivating the p38 mitogen-activated protein kinase pathway

**DOI:** 10.1080/21655979.2022.2056814

**Published:** 2022-03-25

**Authors:** Yifan Zhang, Ruiqi Zhang, Lan Lu, Na Zhou, Xiaoyan Lv, Xin Wang, Zhanbin Feng

**Affiliations:** a Department of cardiovascular medicine, Ninth Hospital of Xi’an, Xi’an City, Shanxi Province, China; b Department of cardiovascular medicine, The First Affiliated Hospital of Xi’an Jiao Tong University, Xi’an City, Shanxi Province, China

**Keywords:** LOX-1, alcoholic cardiomyopathy, P38MAPK, cardiac fibrosis

## Abstract

LOX-1 triggers myocardial fibrosis, but its roles and mechanisms in alcoholic cardiomyopathy and the involvement of the downstream signaling pathways had not been fully reported. We planned to explore how LOX-1 facilitated myocardial fibrosis in alcoholic cardiomyopathy. The in vitro and in vivo alcoholic cardiomyopathy model was established by alcohol treatment to rats’ cardiac fibroblasts and rats, respectively. Masson staining was conducted to observe the collagen deposition and the IHC assay was executed to evaluate the contents of collagen I and III in vitro and in vivo. The cardiac tissues were also observed under TEM and the cardiac function of rats was evaluated using UCG. The expression levels of LOX-1 and P38MAPK in cardiac fibroblasts and tissues at both mRNA and protein levels were analyzed by RT-qPCR and western blot, respectively. Alcohol treatment could trigger collagen deposition, cell hypertrophy, fibrotic changes and increased the expression levels of LOX-1 and P38MAPK both in vivo and in vitro. It also deteriorated the cardiac function of rats in vivo. Overexpression of LOX-1 in vitro could aggravate the fibrotic changes while knockdown of LOX-1 ameliorated the fibrotic effects of alcohol treatment both in vitro and in vivo such as reduction of collagen deposition, relief of cell hypertrophy and inactivation of the P38MAPK signaling pathway. We concluded that knockdown of LOX-1 exerted anti-fibrotic effects via inhibiting P38MAPK signaling in alcoholic cardiomyopathy both in vitro and in vivo. Our findings highlighted that LOX-1 could become a potential therapeutic target in the treatment of alcoholic cardiomyopathy.

## Graphical Abstract

**Figure:**
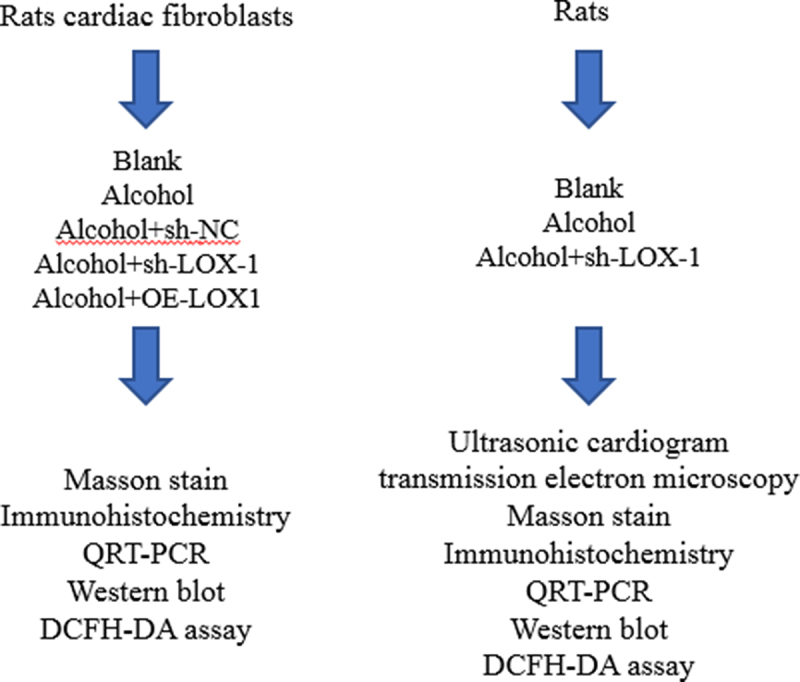

## Introduction

The alcoholic cardiomyopathy is a common form of heart damage induced by the excessive long-term consumption of alcohol and its incidence is still rising [1]. Alcohol can trigger myocardial cellular degeneration, hypertrophy, remodeling, chamber dilatation and heart failure [2]. Cardiac fibrosis is detected in alcoholic cardiomyopathy patients and it is the crucial factor that contributes to the reduction in contractility and arrhythmia [3]. In addition, the initiation and development of alcoholic cardiomyopathy are related to the activation of the molecular pathway. Research has reported that activation of the p38 MAPK/CREB pathway and the renin-angiotensin system is involved in the development of alcoholic cardiomyopathy [4]. Alcohol can also trigger cell damage and ROS production via the p38 MAPK pathway [5]. Currently, the molecular mechanism and pathogenesis of alcoholic cardiomyopathy are still not fully understood and the effective treatment of alcoholic cardiomyopathy is still required.

Lectin-like oxidized low-density lipoprotein-1 (LOX-1) was identified in 1997 and it existed in cardiomyocytes, vascular smooth muscle cells and fibroblasts [6]. It can be activated transcriptionally and translationally by inflammatory cytokines and oxidative stress to exert certain roles in cardiovascular diseases [7]. It is the main receptor of oxidized low-density lipoprotein (oxLDL) [8] and it can activate different pathways such as P38MAPK and NF-κB [9,10]. Besides, it upregulates or activates the ROS-related signaling pathways such as the NAPDH pathway to control cellular oxidative stress response [11]. On the one hand, activation of NAPDH by LOX-1 can accelerate the production of ROS; on the other hand, excessive production of ROS leads to the upregulation of LOX-1 expression [12]. Also, overexpression of LOX-1 stimulates the p38MAPK pathway to boost the expressions of metalloproteinase and adhesion molecules to accelerate myocardial fibrosis [12]. Furthermore, Mehta et al have reported that angiotensin II enhances LOX-1 expression via interaction with angiotensin I receptor and the high expression of LOX-1 can also upregulate the expression of angiotensin I receptor [13]. This process triggers the oxidative stress response and promotes the proliferation of cardiac fibroblasts and collagen synthesis [13]. In contrast, the application of LOX-1 inhibitor reduces the secretion of inflammatory factors and dropped the expression of angiotensin I receptor to ameliorate myocardial hypertrophy and collagen accumulation [14]. In atherosclerosis, LOX-1 expression is positively related to ROS production, autophagy, and immune responses [15]. However, currently, the roles of LOX-1 in alcoholic cardiomyopathy and the involvement of the downstream effector molecules remain unclear.

Therefore, in this research, we plan to overexpress or knock down LOX-1 to explore its effects in alcoholic cardiomyopathy both in vitro and in vivo to provide new insights and the potential therapeutic target for the treatment of alcoholic cardiomyopathy.

## Material & methods

### Cells

The rat cardiac fibroblasts were ordered from Procell (China) and cultured in high glucose DMEM with 10% FBS, 10 µM 5-aza-2ʹ- deoxycytidine and antibiotics (Procell, China) at 37°C in 5% CO2. The cells were divided into 5 groups (Blank, alcohol, alcohol+sh-NC, alcohol+sh-LOX-1 and alcohol+OE-LOX-1). All the groups of cells except the Blank group were incubated with 200 mmol/L alcohol for 1 day before analysis to trigger alcoholic cardiomyopathy *in vitro*.

### Transfection

This research was approved by the Animal Care Committee and the Ethical Committee of Ninth Hospital of Xi’an and obeyed the NIH guidelines. To knock down LOX-1 before alcohol treatment, 3 shRNA and a negative control shRNA was purchased from Genepharma (China). There sequences were: sh-NC: 5’-CCGG ACG ACT GAT AGA GCG ATG CGA CTCGAG TCG CAT CGC TCT ATC AGT CGT TTTTG-3’, 3’-TGC TGA CTA TCT CGC TAC GCT GAGCTC AGC GTA GCG AGA TAG TAC GCA AAAAACTTAA −5’. sh-LOX-1-1: 5’-CCGG AAA ACA ACA GTT AAA GGT CTT CTCGAG AAG ACC TTT AAC TGT TGT TTT TTTTG-3’, 3’-TTT TGT TGT CAA TTT CAA GAA GAGCTC TTC TGG AAA TTG ACA ACA AAA AAAAACTTAA-5’. sh-LOX-1-2: 5’-CCGG TGT TTT ACA CCC AAG AAA GGA CTCGAG TCC TTT CTT GGG TGT AAA ACA TTTTG-3’, 3’-ACA AAA TGT GGG TTC TTT CCT GAGCTC AGG AAA GAA CCC ACA TTT TGT AAAAACTTAA-5’. sh-LOX-1-3: 5’-CCGG ACT TTT GAT ACA AAG ATG CCA CTCGAG TGG CAT CTT TGT ATC AAA AGT TTTTG-3’, 3’-TGA AAA CTA TGT TTC TAC GGT GAGCTC ACC GTA.

And the sequences for LOX-1 overexpression was also designed by Genepharma and transfected into the cardiac fibroblast of the alcohol+OE-LOX-1 group. The transfection process was conducted with Lipofectamine 3000 (Invitrogen, USA). The cells were observed under HT7700 microscopy (Hitachi, Japan).

### Rats

A total of 25 two-month-old healthy male SD rats was purchased (Limibio, China) and 24 of them were randomly divided into three groups (Blank, alcohol, and alcohol+sh-LOX-1). All rats were kept in 25°C and 50% humidity environment with a normal diet and ad libitum under a 12 h light/dark cycle for 2 weeks. The in vivo knockdown of LOX-1 in the rats of the alcohol+sh-LOX-1 group was performed by tail vein injection of the adenovirus vectors containing shRNA for LOX-1 knockdown and the sequence was designed by Genepharma (China). The rats in the blank group were fed normally for 16 weeks [16]. The rats in the alcohol and alcohol+sh-LOX-1 groups were intragastrically administrated with 50% alcohol once a day and the water was replaced by alcohol (1^st^ week: 6 ml/kg/day; 2^nd^ week: 8 ml/kg/day; 3^rd^ week: 10 ml/kg/day; 4^th^-16^th^ weeks: 12 ml/kg/day for 2 times) to induce alcoholic cardiomyopathy [17]. 16 weeks later, the rats were sent for ultrasonic cardiogram (UCG) detection. At last, the rats were sacrificed by euthanasia using isoflurane inhalation and the tissues were collected and analyzed.

### UCG

The cardiac function of rats was checked using the Vevo 3100 Preclinical Imaging System (Fuji, Japan) and MX probe at 15 MHz after the rats were paralyzed using isoflurane via the Vevo Anesthesia System (Fuji, Japan) and the heart rate (HR), left ventricular end-systolic dimension (LVESD), left ventricular end-diastolic dimension (LVEDD), left ventricular end-systolic volume (LVESV), left ventricular end-diastolic volume (LVEDV), stroke volume (SV), ejection fraction (EF) value, fractional shortening (FS) value, cardiac output (CO), left ventricle mass (LVM), left ventricular systolic anterior wall thickness (LVAWs), left ventricular diastolic anterior wall thickness (LVAWd), left ventricular systolic posterior wall thickness (LVPWs) and left ventricular diastolic posterior wall thickness (LVPWd) values were obtained as previously described [18,19].

### Masson staining and immunohistochemistry assay

The samples were fixed and sliced. After deparaffinization and rehydration, the Masson staining kit (Jonin, China) was applied for Masson staining to evaluate the collagen deposition in tissues. The collagen would show blue color while the muscle fiber would present red color. And the Collagen I IHC kit and Collagen III IHC kit (Biolad, China) were also selected according to the manufacturer’s protocols for immunohistochemical detection of collagen I and III in cells and tissues respectively.

### RT-qPCR

The Total RNA Extraction Kit (Solarbio, China) was bought to extract RNA. The reverse transcription was executed using the Hifair III 1st Strand cDNA Synthesis Kit (Yeasen, China). To evaluate the expression levels of LOX-1, p38MAPK and β-actin, the RT-qPCR process was executed with the QuantStudio 7 Pro System (ABI, USA) using the SYBR Green Realtime PCR Master Mix (Solarbio, China). The primer sequences were: LOX-1: forward 5ʹ-CTTGTTACGGACTTACTCGG-3ʹ; reverse 5ʹ-CACTCTATGTATGGCAGCTTA-3ʹ, p38MAPK: forward 5ʹ-GTGCCCGACAGATACCAGAA-3ʹ; reverse, 5ʹ-CAGACGCAACGCTCGGTAGG-3ʹ, and β-actin forward 5ʹ-CACGAAACTACCTGCAACTCC-3ʹ; reverse 5ʹ- CATACTCCTGCATGCTGATC-3ʹ. The relative expression levels were normalized with β-actin by the 2^−ΔΔCt^ method.

### Western blot

Proteins were extracted with Protein Extraction Kit (Solarbio, China) and their concentrations were checked using BCA Kit (Solarbio, China). After being separated using SDS-PAGE (Bio-rad, USA), the proteins were transferred to the PVDF membrane (Merck, USA). After blocking, the samples were treated with primary antibodies against LOX-1 (1:1000, ab60178), p-p38MAPK (1:1000, ab4822), p38MAPK (1:1000, ab170099) and β-actin (1:500, ab6276) (Abcam, USA) at 4°C for 12 h. After being rinsed with TBST (Solarbio, China), the secondary HRP-conjugated antibody was added and incubated for 1 h. Next, after TBST washing, the bands were visualized using the DAB kit (Solarbio, China). The relative expression levels of proteins were obtained via Image J 1.53 f (NIH, USA).

### DCFH-DA assay

The ROS levels in cells and tissues were measured using the DCFDA-DA assay kit (Biolab, China) following the instructions. In detail, cells were seeded in 96-well plates at 1 × 10^4^ cells/well in 100 μL of media for 12 h and washed with 1 × buffer and maintained in 100 μL of 1 × buffer. 20 mM s DCFDA solution was added to each well and incubated for 1 h at 37 C. The cardiac tissues were smashed and lyzed, after washed with 1 × buffer and maintained in 100 μL of 1 × buffer, 20 mM DCFDA solution was added and incubated for 1 h at 37  C. The fluorescence signal was observed under the CKX53 microscope (Olympus, Japan).

### Statistical analysis

The experiments were performed in triplicate. Data were expressed as mean ± SD. GraphPad Prism 8.3.0 (GraphPad LLC, USA) was used for analyses and plotting. The differences were checked using the independent sample t-test for comparison between two groups or one-way analysis of variance (ANOVA) followed by Tukey’s post hoc test for comparison among multiple groups. *P* < 0.05 was recognized as statistically significant.

## Results

### Knockdown of LOX-1 ameliorates myocardial fibrosis in alcohol-treated rats’ cardiac fibroblasts

**Figure 1.**
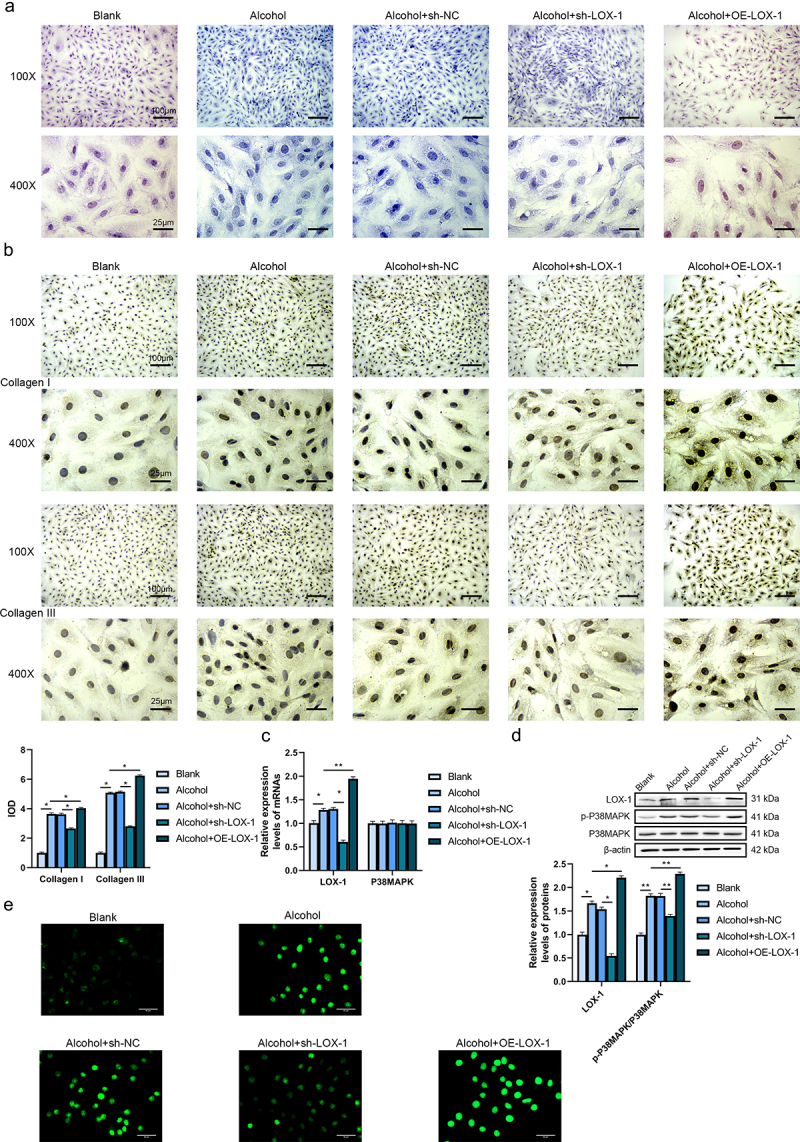
Knockdown of LOX-1 ameliorates myocardial fibrosis in alcohol-treated rats’ cardiac fibroblasts. (a) Collagen deposition in 5 groups of rats’ cardiac fibroblasts was shown by Masson staining under 100X and 400X magnification. Scale bars: 100 μm and 25 μm. (b) The levels of collagen I and III were detected using IHC under 100X and 400X magnification. Scale bars: 100 μm and 25 μm. (c) The relative expression levels of mRNAs in cardiac fibroblasts were analyzed by RT-qPCR. (d) The relative expression levels and phosphorylation levels of proteins in cardiac fibroblasts were assessed by western blot. (e) The ROS level was detected using DCFH-DA assay. Scale bars: 40 μm.*: *P* < 0.05 and **: *P* < 0.01.

In this research, we plan to explore the roles of LOX-1 in alcoholic cardiomyopathy both in vitro and in vivo. After verification of the transfection efficiency using RT-qPCR and western blot assays, the shRNA with the highest knockdown effects was selected to be applied in the subsequent experiments. (Supplementary Fig S1A-B). The results of the Masson staining (Figure 1a) presented that the rats’ cardiac fibroblasts in the alcohol group showed a higher level of collagen deposition, moderate cell hypertrophy, enlarged nucleus and disorganized cell arrangement in contrast to the blank group. Compared with the alcohol+sh-NC group, the cells in the alcohol+sh-LOX-1 presented fewer contents of collagens whereas overexpression of LOX-1 triggers stronger fibrotic influences and more collagen deposition in the cells of the alcohol+OE-LOX-1 group compared with the alcohol group. Besides, the results of IHC reported the elevated expressions of collagen I and III in the alcohol group compared with the blank group which was revealed by the enhanced integrated optical densities (IOD). Knockdown of LOX-1 reduced the levels of collagens while overexpression of LOX-1 could boost the productions of collagen I and III (Figure 1b). Therefore, the expression level of LOX-1 could affect the collagen deposition of rats’ cardiac fibroblasts.

To investigate the action mechanism of LOX-1 in vitro, RT-qPCR and western blot assays were conducted. The results of RT-qPCR reported that alcohol treatment increased the expression level of LOX-1 mRNA but did not change the level of p38MAPK mRNA in the cells of the alcohol group compared with the blank group. Also, knockdown of LOX-1 and overexpression of LOX-1 did not disturb the expression level of p38MAPK mRNA compared with the alcohol+sh-NC group or the alcohol group (Figure 1c). And the results of the western blot showed that alcohol treatment boosted the expression level of LOX-1 and the phosphorylation level of p38MAPK. Knockdown of LOX-1 could inhibit the phosphorylation of p38MAPK relative to the alcohol+sh-NC group whereas overexpression of LOX-1 increased the phosphorylation level of p38MAPK (Figure 1d). In addition to this, the ROS level was detected with DCFH-DA assay and it was indicated that alcohol treatment upregulated the ROS level, knockdown of LOX-1 could reduce the ROS level after alcohol treatment while overexpression of LOX-1 dramatically raised the ROS level (Figure 1e). These results above revealed that knockdown of LOX-1 could retard the fibrotic process caused by alcohol treatment via activating the P38MAPK pathway and increasing ROS level whereas overexpression of LOX-1 could exacerbate the alcohol-induced cardiac fibrosis in vitro.

### Knockdown of LOX-1 ameliorates myocardial fibrosis in alcoholic cardiomyopathy rats in vivo

**Figure 2.**
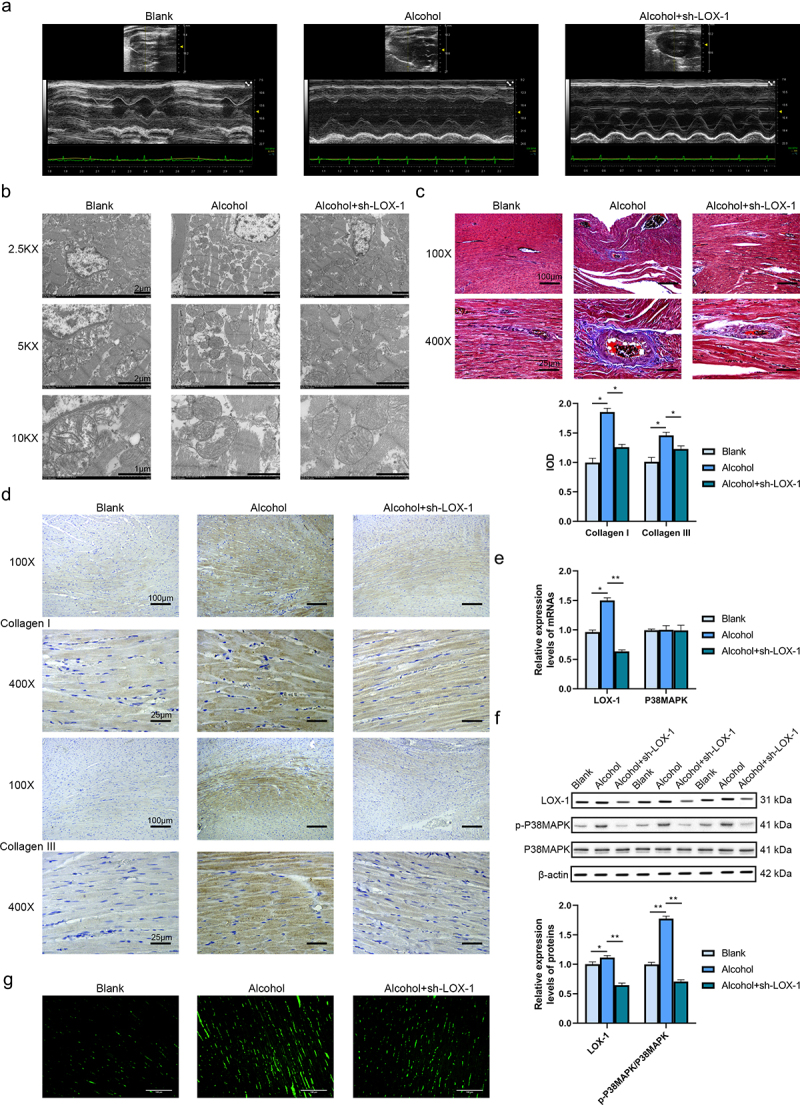
Knockdown of LOX-1 ameliorates myocardial fibrosis in alcoholic cardiomyopathy rats in vivo. (a) The UCG images of three groups of rats. (b) The cardiac tissues were observed by TEM under 2.5KX, 5KX and 10KX magnification. Scale bars: 2 μm, 2 μm and 1 μm. (c) Collagen deposition in rats’ cardiac tissues was shown by Masson staining under 100X and 400X magnification. Scale bars: 100 μm and 25 μm. (d) The levels of collagen I and III were detected using IHC under 100X and 400X magnification. Scale bars: 100 μm and 25 μm. (e) The relative expression levels of mRNAs in rats’ cardiac tissues were analyzed by RT-qPCR. (f) The relative expression levels and phosphorylation levels of proteins in rats’ cardiac tissues were assessed by western blot. (g) The ROS level was detected using DCFH-DA assay. Scale bars: 100 μm.*: *P* < 0.05 and **: *P* < 0.01.

UCG was performed to check the cardiac function of three groups of rats (Blank, alcohol and alcohol+sh-LOX-1) (Figure 2a and Table 1). The results indicated that the rats in the alcohol group had significantly lower HR, EF and FS, and higher LVESD, LVESV, LVM in contrast with the rats in the blank group. Besides, alcohol treatment did not significantly affect the LVEDD, LVEDV, SV and CO of the rats. Compared with the alcohol group, the rats in the alcohol+sh-LOX-1 group had significantly higher HR, SV, EF, FS and CO values. There was no significant difference detected in the LVAWs, LVAWd, LVPWs and LVPWd values among these three groups of rats. These findings indicated that alcohol damaged the cardiac function of rats and knockdown of LOX-1 showed beneficial effects against alcohol-induced damages on the rats’ cardiac function.

**Table 1.** The cardiac function index of rats was evaluated by UCG

	Groups		
Cardiac function index	Blank	alcohol	alcohol+sh-LOX-1
HR (BPM)	384.92 ± 16.98	329.71 ± 14.32^a^	367.58 ± 19.76^b^
LVESD (mm)	2.82 ± 0.62	5.39 ± 1.03^a^	3.37 ± 0.95
LVEDD (mm)	7.77 ± 1.36	8.21 ± 2.01	8.03 ± 1.45
LVESV (μL)	30.18 ± 4.41	141.39 ± 10.36	106.78 ± 7.44
LVEDV (μL)	323.66 ± 26.85	365.37 ± 27.96	330.38 ± 24.33
SV (μL)	293.47 ± 20.36	224.59 ± 18.79^a^	267.98 ± 20.79^b^
EF (%)	90.67 ± 8.96	61.34 ± 7.58^a^	81.91 ± 10.23^b^
FS (%)	63.67 ± 6.58	34.67 ± 5.43^a^	54.13 ± 7.11^b^
CO (mL/min)	112.97 ± 14.73	73.93 ± 10.01	98.52 ± 9.34^b^
LVM (mg)	936.86 ± 39.98	996.98 ± 45.07^a^	985.10 ± 47.36
LVAWs (mm)	2.94 ± 0.74	2.01 ± 0.82	2.81 ± 0.67
LVAWd (mm)	1.48 ± 0.59	1.53 ± 0.38	1.52 ± 0.23
LVPWs (mm)	3.39 ± 0.75	2.74 ± 0.67	2.84 ± 0.99
LVPWd (mm)	1.81 ± 0.61	1.70 ± 0.59	1.73 ± 0.56

a: *P* < 0.05 compared with the blank group.

b: *P* < 0.05 compared with the alcohol group.

The cardiac tissues of rats were observed under the transmission electron microscope (TEM), the images (Figure 2b) showed hypertrophic changes in the alcohol group in contrast with the blank group. In addition, compared with the alcohol group, the cells in the alcohol+sh-LOX-1 group presented fewer pathological changes. It was detected that knockdown of LOX-1 reduced the hypertrophic and fibrotic changes and nuclear enlargement. Besides, the results of the Masson staining (Figure 2c) showed that the cardiomyocytes in the alcohol group were hypertrophic, the nucleus was enlarged and the cell arrangement was disorganized compared with the blank group, and there was focal fibrosis detected. In the alcohol+sh-LOX-1 group, a less number of degenerative and hypertrophic cells was detected, the nucleus was smaller and the fibrotic changes were alleviated in contrast to the alcohol group. Next, the contents of collagen I and III in cardiac tissues of three groups of rats were assessed with IHC (Figure 2d). It was found that the IOD of collagen I and III in the alcohol group were increased relative to the blank group while knockdown of LOX-1 in the alcohol+sh-LOX-1 group attenuated the collagen production compared with the alcohol group. Therefore, knockdown of LOX-1 could ameliorate the alcohol-induced cardiac fibrosis in rats.

To investigate the action mechanism of LOX-1 in vivo, RT-qPCR and western blot assays were executed. The results reported that the rats’ tissues of the alcohol group showed a higher expression level of LOX-1 mRNA. After the knockdown of LOX-1 using shRNA, the expression level of p38MAPK mRNA did not change in contrast to the alcohol group (Figure 2e). And the results of the western blot indicated that knockdown of LOX-1 reduced the phosphorylation level of p38MAPK (figure 2f). Last but not least, alcohol treatment increased the ROS level and knockdown of LOX-1 could reduce the ROS level after alcohol treatment (Figure 2g). These results above indicated that knockdown of LOX-1 relieved alcohol-induced damages to the rats’ hearts in vivo via inhibiting P38MAPK signaling.

## Discussion

This study exhibited the roles of LOX-1 on alcoholic cardiomyopathy both in vitro and in vivo. Our results presented that LOX-1 significantly accelerated ethanol-induced cardiac fibrosis. More importantly, knockdown of LOX-1 exerted beneficial effects by reducing cardiac damages in alcohol-treated cardiac fibroblasts and rats via inhibition of the p38MAPK pathway. These outcomes highlighted that LOX-1 might represent a novel therapeutic target in the treatment of alcoholic cardiomyopathy.

Acute and chronic consumption of alcohol triggers systemic toxic effects on the human body. The liver is the most affected organ, since ethanol is mostly metabolized there, but gastrointestinal, central, and peripheral nervous systems; the heart and vascular system; endocrinological systems; nutrition; and musculoskeletal systems are all affected. In addition, ethanol is an immunosuppressive drug that is pro-inflammatory and pro-oncogenic [2]. The heart is an attacking organ of ethanol. The process of pharmacokinetic and metabolism of ethanol in rats was similar to humans [20,21] and long-time and excessive alcohol consumption triggers cardiac fibrosis, which is a common pathological change detected in most forms of myocardial damage and heart diseases [2] in both of human and rats models. This pathology involves excessive deposition of extracellular matrix (ECM) proteins produced by cardiac fibroblasts which subsequently led to the reduction of tissues compliance [22]. Latter, the cardiac ejection function would be injured, atherosclerosis, cardiac remodeling and accelerated progression to heart failure would also occur [23]. In our study, it was found that alcohol treatment could cause fibrosis both in vitro and in vivo such as cell morphological changes, increased collagen deposition, increased the expression levels of LOX-1 and activated p38MAPK, and reduced heart function in rats. Our results were consistent with Wang et al’s paper which proposed that in the alcohol-treated rats, cardiac dysfunction and morphological changes and increased fibrosis were detected [24]. Therefore, research focusing on cardiac fibroblasts and deposition of ECM should be continuously conducted to enhance the molecular therapeutic method against alcoholic cardiomyopathy.

LOX-1 is a transmembrane protein with its proform of 50 kDa and mature form of 31 kDa in endothelial cells and it has been reported to play promotive roles in atherosclerosis and vasculopathy via LOX-1-ox-LDL interaction [25]. It served as a diagnostic marker and treatment target for treating cardiovascular diseases. Currently, most of the published articles focused on its roles in atherosclerosis and little evidence could be found about its effects on cardiac fibrosis. Our results reported that overexpression of LOX-1 in vitro enhanced collagen I and III deposition and hypertrophy of cardiac fibroblasts after alcohol treatment whereas knockdown of LOX-1 by shRNA showed opposite effects. In addition to this, knockdown of LOX-1 could also ameliorate the alcohol-induced injury and enhance the cardiac function of rats *in vivo*. Our results were similar to Lu et al’s finding which also showed that deletion of LOX-1 could relieve cardiac hypertrophy and fibrosis-related collagen accumulation after chronic ischemia in mice [26]. We also found that LOX-1 contributed to the ROS production and knockdown of LOX-1 could reduce ROS production both in vitro and in vivo. This was consistent with Li et al’s findings [27]. Furthermore, abrogation of LOX-1 also exerted protective roles against doxorubicin-induced myocardial inflammation, fibrosis and degeneration [28]. Therefore, LOX-1 owned fibrotic roles in cardiovascular disease.

In our study, it was found that overexpression of LOX-1 could increase the phosphorylation level of P38MAPK in vitro whereas knockdown of LOX-1 reduced its phosphorylation level both in vitro and in vivo. This was consistent with the previous study [29]. p38MAPK is a group of kinases activated by stress and inflammation that modulates cardiac fibroblast function, such as inflammatory responses, remodeling, ECM production and cardiomyocyte hypertrophy [30]. It could be activated by ethanol and ROS production was involved in this process [31]. Kojonazarov et al have reported that blocking of the P38MAPK pathway improved heart function and suppressed hypertrophy and fibrosis [32]. The activated p38 pathway contributed to cardiac fibrosis [33]. Another evidence also exhibited that the activated p38MAPK pathway contributed to the progression of myocardial fibrosis [34]. Kimoto et al presented that the activated P38MAPK pathway could increase collagen production [35]. Zhao et al also pointed out that inhibition of p38MAPK significantly attenuated cardiac hypertrophy and fibrosis [36].

## Conclusion

In conclusion, our in vivo and in vitro studies demonstrated that knockdown of LOX-1 could show anti-fibrotic roles and protective effects to the heart potentially via suppressing the P38MAPK signaling. The main limitations of this study were the lack of using pathway inhibitors to perform the rescue experiments. Furthermore, the investigation of more upstream and downstream molecules involved in the fibrotic roles of LOX-1 and P38MAPK would be conducted in the future. The current findings provide insights into the molecular action mechanisms of LOX-1 in cardiac fibrosis and alcoholic cardiomyopathy.

## Supplementary Material

Supplemental Material

## Data Availability

The datasets used during the present study are available from the corresponding author upon reasonable request.
