# Supplementary figures and images for: Knockdown of lectin-like oxidized low-density lipoprotein-1 ameliorates alcoholic cardiomyopathy via inactivating the p38 mitogen-activated protein kinase pathway
Source: Bioengineered. 2022 Mar 25;13(4):8926–36. doi: 10.1080/21655979.2022.2056814 (PMC9161863; doi:10.1080/21655979.2022.2056814)

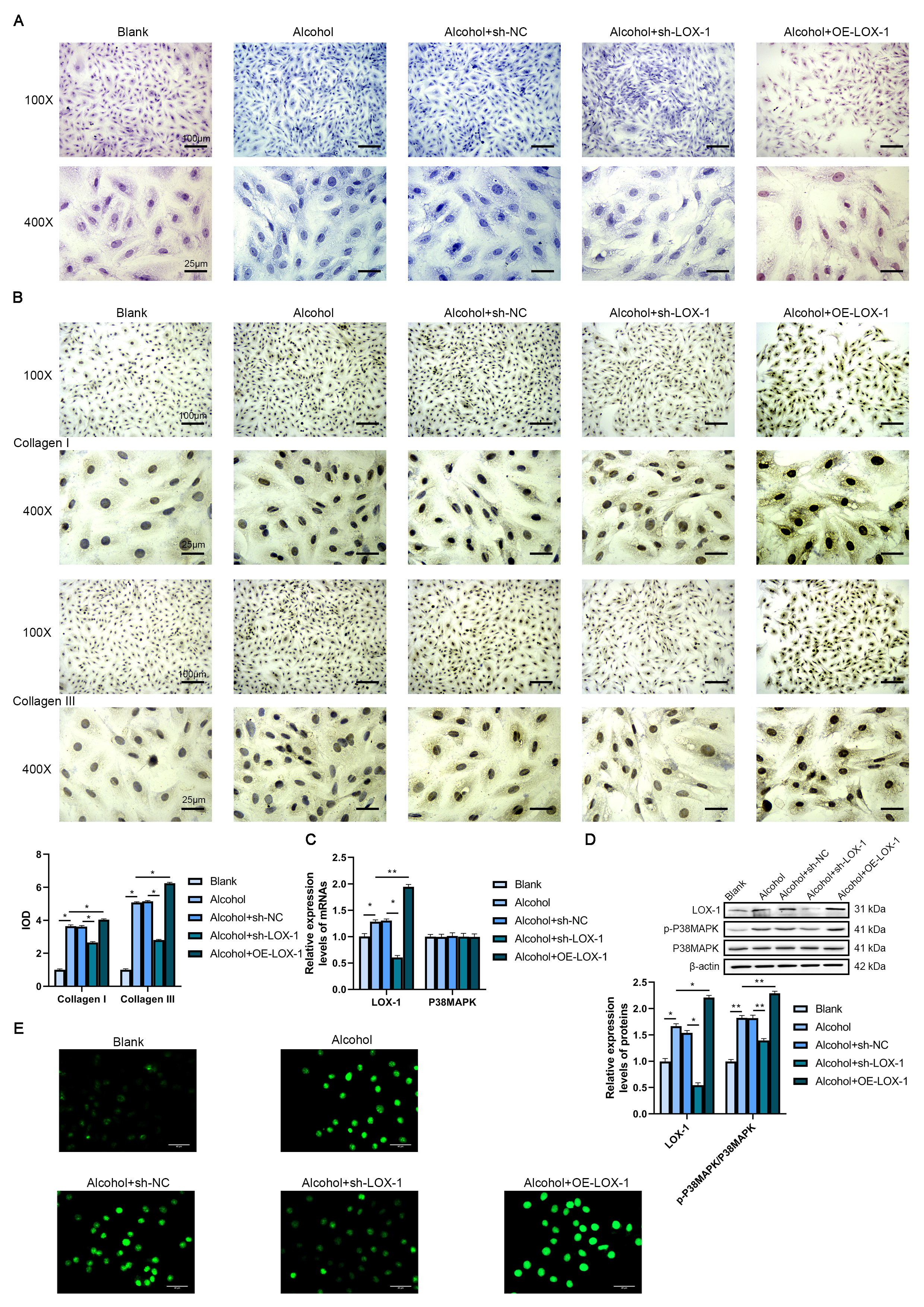

Supplement: Supplemental Material [file KBIE_A_2056814_SM9511.tif]
